# Ferromagnetic CaRuO_3_

**DOI:** 10.1038/srep03877

**Published:** 2014-01-27

**Authors:** Shivendra Tripathi, Rakesh Rana, Sanjay Kumar, Parul Pandey, R. S. Singh, D. S. Rana

**Affiliations:** 1 Indian Institute of Science Education and Research (IISER) Bhopal, M.P.–462023, INDIA

## Abstract

The non-magnetic and non-Fermi-liquid CaRuO_3_ is the iso-structural analog of the ferromagnetic (FM) and Fermi-liquid SrRuO_3_. We show that an FM order in the orthorhombic CaRuO_3_ can be established by the means of tensile epitaxial strain. The structural and magnetic property correlations in the CaRuO_3_ films formed on SrTiO_3_ (100) substrate establish a scaling relation between the FM moment and the tensile strain. The strain dependent crossover from non-magnetic to FM CaRuO_3_ was observed to be associated with switching of non-Fermi liquid to Fermi-liquid behavior. The intrinsic nature of this strain-induced FM order manifests in the Hall resistivity too; the anomalous Hall component realizes in FM tensile-strained CaRuO_3_ films on SrTiO_3_ (100) whereas the non-magnetic compressive-strained films on LaAlO_3_ (100) exhibit only the ordinary Hall effect. These observations of an elusive FM order are consistent with the theoretical predictions of scaling of the tensile epitaxial strain and the magnetic order in tensile CaRuO_3_. We further establish that the tensile strain is more efficient than the chemical route to induce FM order in CaRuO_3_.

Among various 4d transition metal oxides, the metallic SrRuO_3_ is the only system which exhibits a long-range ferromagnetic order with a Curie temperature of 165 K^1^,^2^,^3^. Attributes of a large magnetic moment and the metallic behavior of SrRuO_3_ make it as one of the most suitable materials as ferromagnetic metal electrodes in the spintronic devices based on spin-polarized tunnel junctions^4^. Owing to various fundamental and technological interests in SrRuO_3_, a variety of studies have been devoted on this compound in the recent past. However, the large magnetic coercivity of SrRuO_3_ is a drawback in context of the use of its magnetic order in magnetic devices. In this regard, CaRuO_3_ is a natural alternative choice as it is iso-structural and iso-electronic with SrRuO_3_ and is metallic down to low temperatures^5^. It also exhibits a wide variety of properties such as non-fermi liquid behaviour^5^, magnetic quantum criticality^6^, pressure induced post-perovskite structure^7^, etc. However, contrary to expectations the CaRuO_3_ does not exhibit a long-range magnetic ordering. Though it is a known metallic paramagnet^8^,^9^, some studies have indicated that it exhibit antiferromagnetic order with T_N_ ~ 110 K^2^. Its magnetic state, therefore, is still debated and is far from established. One agreement that has been reached among various researchers is that the CaRuO_3_ is on the verge of establishing magnetic correlations^2^,^10^. In ABO_3_ structure, the difference in ionic radius of A-site cations Sr^2+^ (~1.31 Å) and Ca^2+^ (~1.12 Å) is responsible for the difference in the ground state of SrRuO_3_ and CaRuO_3_^11^,^12^. Owing to this reason, there is a definite interest in understanding and manipulating the magnetic ground state of CaRuO_3_ by the means of chemical substitution, disorder and strain with an aim to obtain ferromagnetic order as in its counterpart SrRuO_3_.

The CaRuO_3_ is a metal with a GdFeO_3_ type orthorhombic structure (a = 5.541 Å, b = 5.362 Å, and c = 7.686 Å, space group – P*nma*). The central Ca atom is surrounded by corner sharing RuO_6_ octahedra^14^. The distortion of RuO_6_ octahedra affects the Ru-O-Ru bond angles, which consequently affects the electronic and magnetic properties^11^. As per the phase diagram of a class of perovskites, the CaRuO_3_ lies in the close vicinity of quantum critical region which separates FM and Fermi-liquid systems from the antiferromagnetic and non-Fermi liquids. It is established that both CaRuO_3_ and SrRuO_3_ have strikingly similar electronic structure and correlations^11^,^12^. So, the sole reason for difference in their magnetic and electrical properties lies in the structural distortion; it is the size effect which results in larger Ru-O-Ru bond angles for the SrRuO_3_ (see figure 1). Several theoretical studies have suggested the metallic CaRuO_3_ to be on the verge of establishing ferromagnetic phase transition^6^,^13^. A magnetic order in CaRuO_3_ is expected if the Ru-O-Ru bond angles and bond distances can be manipulated by two primary means, namely, chemical substitution at Ca and Ru sites or physical means of epitaxial and uniaxial strains in thin films. He and Cava reported that disorder created by substitution of non-magnetic Ti at Ru site in CaRuO_3_ induces ferromagnetism in the system^6^. Extensive studies on CaRu_1−x_M_x_O_3_, where M (transition metal) is either a magnetic or non-magnetic ion, have shown doping induced discernible modifications of the magnetic and electronic phases^15^,^16^. Amongst various ions, the Cr substitution for Ru has proved the most effective as it induces substantial ferromagnetism in CaRuO_3_^17^,^18^,^19^,^20^, albeit transition from metallic to insulating state.

Understanding and manipulating the magnetic ground state of metallic CaRuO_3_ is one of the key issues in perovskite ruthanates. All above-mentioned methods to induce FM order in CaRuO_3_ involve chemical substitution which disrupts the Ru sub-lattice. Zayak et al theoretically showed that a magnetic order in chemically pure CaRuO_3_ can be established by applying tensile strain and that the magnitude of the induced FM moments scales with the tensile strain^20^. Compressive strain, on the other hand, does not modify the magnetic state. Experimental observations of any such effects in which FM moment is induced as a function of epitaxial strain in single layer phase-pure films are yet to be realized. In the present work, we have deposited the CaRuO_3_ thin films on substrates with lattice constants inducing either the compressive strain or the tensile strain. In this letter, we show that the tensile strain induces a weak FM order in pure CaRuO_3_ thin films and that the magnetic moment scales with the tensile strain, which is commensurate with the theoretical predictions. We further demonstrate that the tensile strained CaRuO_3_ films possess larger magnetic moment compared to that of chemically modified CaRu_0.9_Cr_0.1_O_3_ films.

The CaRuO_3_ films of various thicknesses, in the range of 20–140 nm, were deposited using a 248 nm KrF excimer laser. The parameters for various depositions were: energy density between 1.7–3.3 J/cm^2^, laser pulse frequency - 4 Hz, substrate temperature - 700°C, O_2_ partial pressure - 40 Pa, O_2_ annealing pressure of 1000–1800 Pa. The SrTiO_3_ (100) [STO] substrate (lattice constant ~3.905 Å) with a mismatch of about 2% was chosen to obtain tensile strained CaRuO_3_ (CRO) thin films. Though phase-pure oriented films were formed on BaTiO_3_ (100) [a ~ 3.99 Å] and MgAl_2_O_4_ (100) [a ~ 4.04 Å] substrates, strained films could not be obtained as the lattice mismatch of these substrates with CRO is too large to be accommodated for stability of strained phase. Only films on STO substrate could be stabilized with a reasonable tensile strain. It is known that the CRO films on STO have a tendency for formation of pseudo-heterostructures^28^. The problem with these films is that it is difficult to assign the origin of magnetic moment, if any, to any of the co-existing phases. Hence, it is required to segregate these epitaxial phases and investigate their magnetic properties. We started the usual deposition by varying the energy density and keeping other parameters fixed. The film obtained with energy density of 1.7 J/cm^2^, say CRO-A, possessed two phases as evident from two closely spaced epitaxial reflections in θ-2θ patterns (Fig. 1a). To get rid of one phase in this pseudo-heterostructure, the energy density was increased to 2.3 J/cm^2^. Thus obtained film, say CRO-B, too possessed two epitaxial phases. Finally, films with single homogenous phase were obtained when laser energy density was fixed at 2.0 J/cm^2^. With these optimized parameters, films with thickness of 130 nm and 30 nm, respectively, labeled as CRO-C and CRO-D were deposited. For CaRu_0.9_Cr_0.1_O_3_, the 30 nm and 130 nm films (CRO10-A and CRO10-B, respectively) were deposited with optimized energy density of 2.0 J/cm^2^.

## Results

**Figure 1:**
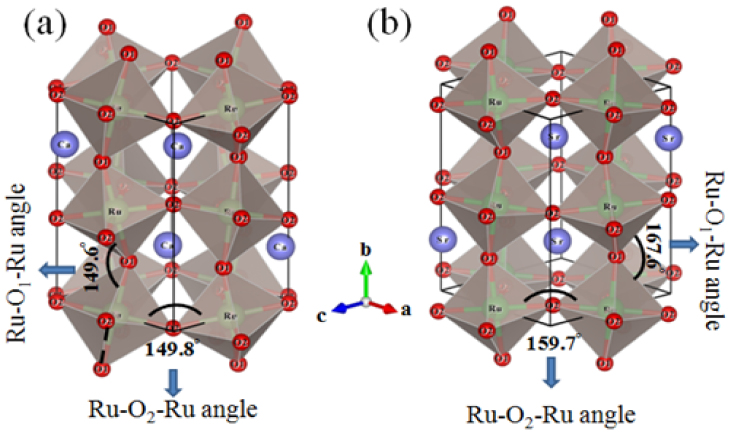
Schematic of the distorted RuO_6_ octahedra for (a) CaRuO_3_ and (b) SrRuO_3_.

**Figure 2:**
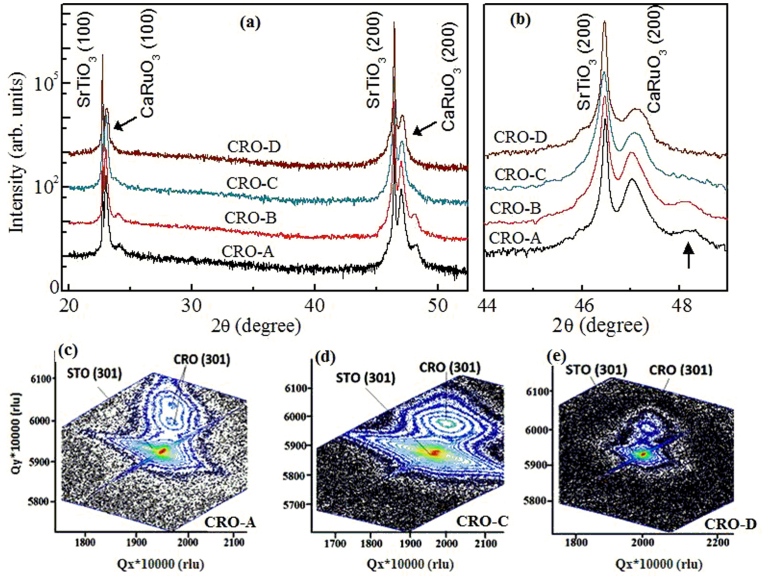
(a) θ-2θ XRD patterns for CRO-A, CRO-B, CRO-C and CRO-D films; (b) (200) peak is magnified in which the arrow film CRO-A and CRO-B indicates another CRO phase. Reciprocal space maps for (c) CRO-A film, (d) CRO-C film and (e) CRO-D film.

Figure 2 (a) shows the θ-2θ x-ray diffraction (XRD) patterns of four representative CaRuO_3_ thin films. The CRO-A and CRO-B films show two closely spaced peaks indicative of two co-existing structures. There is a clear splitting of both (100) and (200) peaks in CRO-A film (Fig. 2a), which arises from co-existing structural polymorphs, namely, fully and/or partial strained orthorhombic and cubic phases of the CRO film. On the other hand, the optimized films, namely, CRO-C and CRO-D showed only one epitaxial reflection suggesting only one structural form for these films. For analyzing the strain states in the films, the reciprocal space maps (RSMs) were acquired around asymmetric (301) peak. Figure 1c–e shows RSMs for CRO-A, CRO-C and CRO-D films around (301) peak. The salient features of these data are: i) similar to that of data in Fig. 2a, these RSM data show that the CRO-A shows dual peaks while the CRO-C and CRO-D films show only one epitaxial peak, ii) the peak of the films do not lie on the pseudomorphic line of the STO substrate implying that none of these films is coherently strained and that the in-plane lattice parameters of the substrate and film are not the same, iii) among the films exhibiting only one peak (i.e., in CRO-C and CRO-D), the out-of plane lattice parameter of 3.876(1) Å for CRO-C film with a thickness of 130 nm decreased to 3.854(1) Å for CRO-D film. This suggests that the tensile strain increases with decreasing thickness, which is further supported by the variations in the in-plane parameters of all these films, as calculated from the RSM data. This is consistent with behavior of tensile strained films; as the out-of-plane lattice constant decreases, the in-plane parameter increases to accommodate the strain. We observed the same as one of the in-plane lattice constant increased from 3.866(1) Å for CRO-C film to 3.883(1) Å for CRO-D film. In CRO-A and CRO-B films, the out-of-plane lattice constant of either of the reflections do not match with that of CRO-C or CRO-D film. Overall, these data suggest that all the films are partially strained film, among which the films with single structural phase exhibit increasing tensile strain with decreasing thickness.

**Figure 3:**
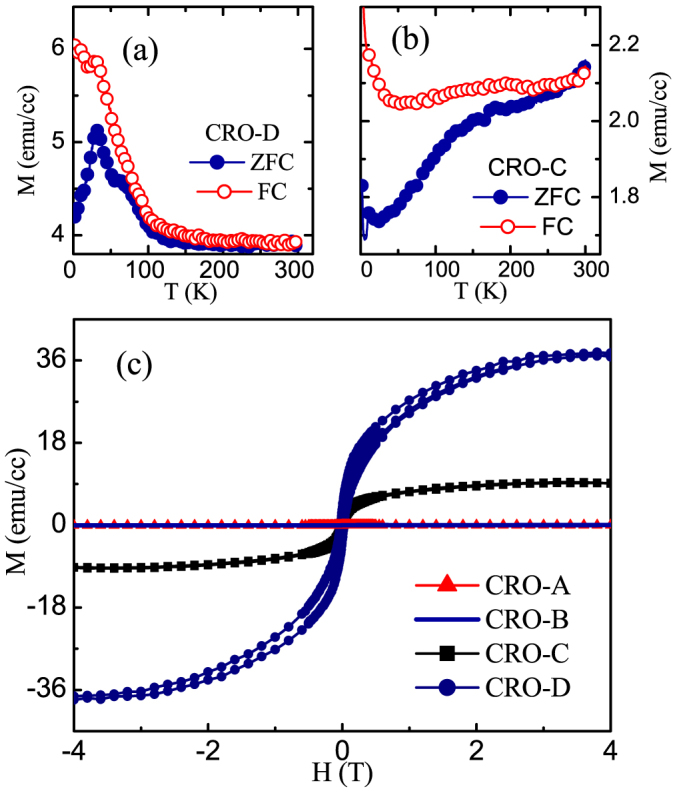
Zero-field-cooled (ZFC) and field-cooled (FC) Magnetization (M) versus temperature plots for (a) CRO-D film, and (b) CRO-C film. (c) Magnetization (M) versus field (H) isotherms at T = 10 K for CRO-A, CRO-B, CRO-C and CRO-D films.

Figure 3a–b shows the zero-field-cooled (ZFC) and field-cooled (FC) magnetization (M) versus temperature (T) data obtained in a field of 500 Oe for all the CaRuO_3_ films. It is seen that the films with only one phase, i.e., CRO-C and CRO-D, exhibit ferromagnetic (FM) like phase transition whereas CRO-A and CRO-B films do not show any indication of FM phase down to low temperatures. In the CRO-C film, the transition temperature is not well defined, but the bifurcation between ZFC and FC curves clearly points towards weak FM phase in this film. The CRO-D film, however, exhibits a pronounced magnetic transition with clearly discernible transition temperature in the vicinity of 100 K. Also, the FC magnetic moment of this film is about 3–4 times more than that of CRO-C film. This suggests that magnetic transition that tends to set in thicker CRO-C film manifests itself more clearly in more strained CRO-D film. To ascertain the hysteretic nature of the FM phase in these films, magnetization versus magnetic field isotherms for all the films were collected at 10 K (Fig. 3c). A linear variation of magnetization with magnetic field for CRO-A and CRO-B films unambiguously confirmed non-magnetic nature of these films. The CRO-C and CRO-D films, whereas, showed a FM like hysteresis which clearly corroborated with their temperature dependent magnetization data for existence of the FM order in these films. Furthermore, the saturation magnetic moment of these films increased with increasing tensile strain, larger for the CRO-D film compare to that for the CRO-C film. Overall, from both the temperature- and field-dependent magnetization data of these magnetic CaRuO_3_ films, it may be inferred that the magnitude of magnetic moment scales with the tensile strain.

**Figure 4:**
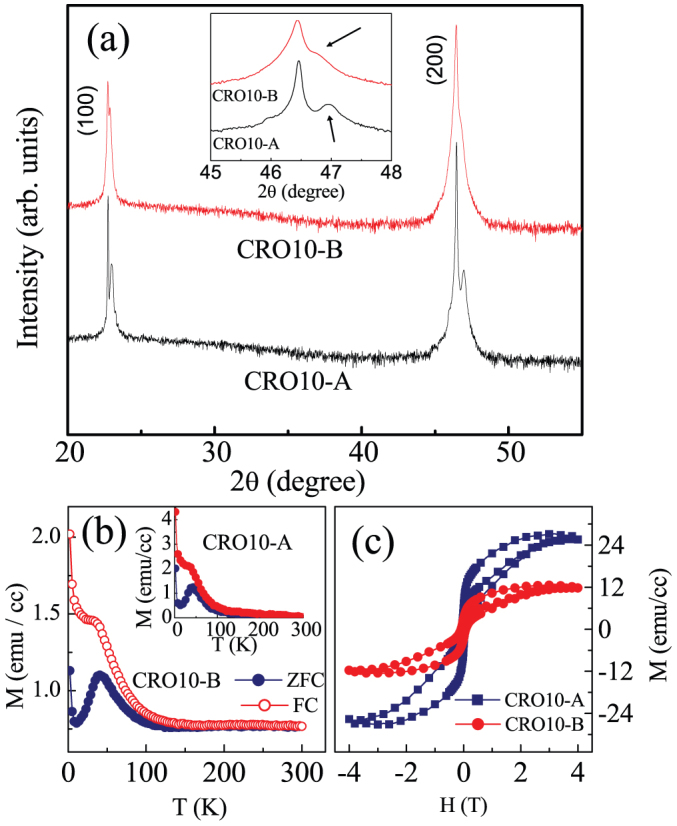
(a) θ-2θ XRD plots for CRO10-A and CRO10-B films. The (200) peak is magnified in the inset figure. Arrows in this figure indicate the (200) peak of CaRuO_3_. (b) Zero-field-cooled (ZFC) and field-cooled (FC) Magnetization (M) versus temperature plots for CRO10-A and CRO10-B films. (c) Magnetization versus field (H) isotherms for same films measured at T = 10 K.

The efficiency of strain in inducing the magnetic moment in CaRuO_3_ was evaluated by comparing the magnetic properties of the strained films and the Cr-modified films. The CaRu_1−x_Cr_x_O_3_ (x = 0–0.3) series of bulk compounds shows a maximum of saturation magnetic moment (M_S_) of ~0.35 *μ_B_/f.u* for x = 0.15^17^. The M_S_ decreases sharply for x > 0.15 and for x < 0.15. In our studies we have deposited films of x = 0.10 for which the M_S_ of the bulk compound is ~0.15 *μ_B_/f.u*^17^. We investigated the structure and magnetization of two weakly FM CaRu_0.9_Cr_0.1_O_3_ films, namely, a 30 nm film (CRO10-A) and a 130 nm film (CRO10-B). Figure 4 show the XRD and the magnetization data of these films. Similar to that in pure CRO films, the out-of-plane lattice constant decreases from 3.886(1) Å to 3.867(1) Å as the film thickness decreases from 130 nm for CRO10-B film to 30 nm for CRO10-A film. A consequent increase in the in-plane lattice parameters, as confirmed by RSM data (not shown here), suggests a larger tensile strain for 30 nm film. The ZFC-FC magnetization versus temperature data shows that both the films exhibit FM transition at around same temperature (Fig. 4b). The magnetization-field isotherms taken at 10 K clearly show that the saturation magnetization increases with the decreasing thickness of film, which implies that the FM moment increases as the tensile increases in Cr-doped films (Fig. 4c). There are two implications of these results, namely, i) the scaling of tensile strain and magnetization is similar to that observed for pure CRO films and ii) magnetic moment of pure strained CRO-D film is marginally large compared to that of Cr-doped strained CRO10-A film. This is surprising because Cr-doping alone has proved most efficient in inducing the FM order in CRO^17^. In present case, the Cr-doped films were strained to same extent as the pure films. Despite this, larger moment in pure films suggests that the tensile epitaxial strain is clearly more efficient than the chemical route to induce magnetic order in otherwise non-magnetic CaRuO_3_.

**Figure 5:**
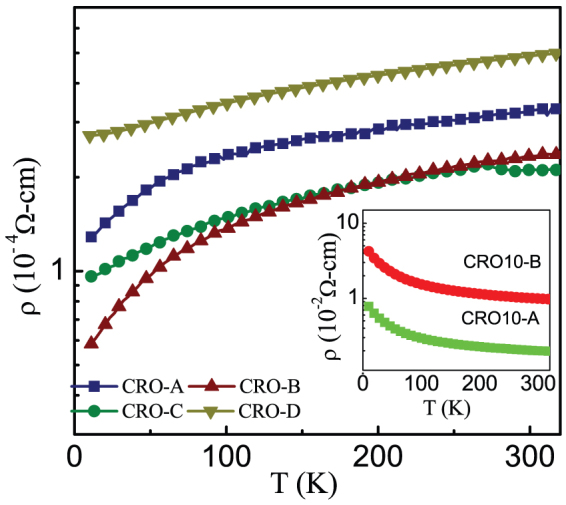
Resistivity (ρ) as a function of temperature (T) data for all the pure CaRuO_3_ films (main panel) and Cr-doped films (inset).

At this point, it is imperative to discuss the Ca/Ru ratio in the CRO films under investigation. In an elaborate study, Rao *et al* performed Rutherford Backscattering experiments on CRO/STO (100) thin films prepared under different conditions and showed that the stoichiometric films exhibit a bulk-like metallic behavior whereas the non-stoichiometric films exhibit semiconducting behavior^21^. In present case, the temperature dependence of electrical resistivity for all films reveals that all the CaRuO_3_ films exhibit metallic behavior (Fig. 5) while the Cr-doped films exhibit semiconducting behavior (see inset of Fig. 5). In both the cases, the resistive behavior is representative of their respective polycrystal bulk counterparts^2^. This suggests a stoichiometric Ca/Ru ratio in the present CaRuO_3_ films. Also it is known that the non-stoichiometric films possess lattice parameters quite different from that of the bulk then the stoichiometric films^21^. We find that a unit cell volume (~231 Å^3^) of the present films is very close to that of the bulk (~228.5 Å^3^). On the other hand, the Ca-rich non-stoichiometric CRO/STO (100) films^20^ possess a significantly larger cell volume of ~242 Å^3^. This clearly suggests that all the films used in the present work possess close to ideal Ca/Ru ratio.

**Figure 6:**
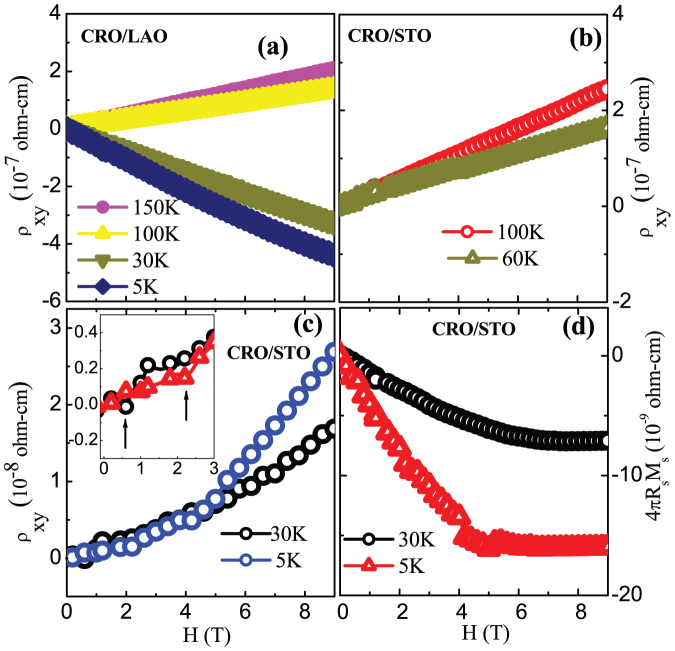
Magnetic field (H) dependent Hall resistivity ρ_xy_ at different temperature for (a) CRO/LAO film (b) High temperature regime of CRO/STO film (c) Low temperature regime of CRO/STO film. Inset shows the non linear behavior of ρ_xy_ for T = 5 K and 30 K in the vicinity of H ~ 2 T (d) Anomalous Hall resistivity (4πR_s_M_s_) as a function of magnetic field for CRO/STO film.

Hall resistivity measurements represent a reliable method of determining the carrier concentration, the sign of the charge carriers in conducting systems and the magnetic ground state^22^. The Hall resistivity (ρ_xy_) in is commonly arises from the ordinary Hall effect (OHE) and the anomalous Hall effect (AHE), as given by the relation^23^,^24^

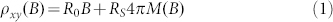
where B is the magnetic field. First and second terms on right-hand side originate from ordinary and anomalous Hall effects (*ρ_OHE_* and *ρ_AHE_*), respectively. R_0_ is coefficient of *ρ_OHE_* which depends on carrier concentration via relation R_0_ = 1/(nq) [n - carrier density, and q - carrier charge]. The R_s_ is the coefficient of *ρ_AHE_*, which arises from the sample magnetization. Also, the *ρ_AHE_* scales with the magnetic moment and has its origin in spin-orbit interactions. The variation of *ρ_OHE_* with magnetic field is linear as B ~ μ_o_H. Any nonlinearity in *ρ_xy_* as a function of magnetic field is attributed to the emergence of AHE. The *ρ_AHE_* is directly proportional to sample magnetization. Hence, the nonlinear AHE contribution to the *ρ_xy_* originates from the ferromagnetic order in the system. In the low-field regime, the contribution of OHE is generally negligible compared to the AHE. Hence, a non-zero value of *ρ_AHE_* in low field regime points towards ferromagnetic order in the system^23^,^24^. To verify the magnetic ground state via anomalous Hall component in our samples, Hall measurement were performed on two CaRuO_3_ films, namely, a non-magnetic compressive-strained film on LaAlO_3_ (100) substrate [CRO/LAO] and a magnetic tensile-strained film on SrTiO_3_ (100) substrate [CRO/STO]. As seen in Figure 6, the CRO/LAO film exhibits linear variation of *ρ_xy_* with the applied field. Also, there is switching in sign of charge carriers at about 50 K, however, albeit absence of any non-linear component (Fig. 6a). This is in agreement with existing report^25^. In the magnetic CRO/STO film, however, there is a distinctive non-linear behaviour in *ρ_xy_* which arises from AHE having its origin in ferromagnetic ground state (Fig. 6b–c). Separation of OHE and AHE from the *ρ_xy_* is done in the following way. The high-field slope of *ρ_xy_* versus magnetic-field provides R_0_ as the high-field magnetization gets saturated (dM/dH ~ 0) and this contribution of *ρ_xy_* depicts the behavioral trend of OHE. Furthermore, the sign of R_0_ suggests the type of charge carriers, *i.e.*, the holes or the electrons. The anomalous component is determined by the extrapolation of high-field linear *ρ_xy_* to H = 0. This extrapolated non-zero *ρ_xy_* at H = 0 is the contribution from the AHE. We observed a distinct non-linear behavior in *ρ_xy_* vs magnetic field below T = 60 K which manifests as a discernible “kink” in the vicinity of H = 2 T (see inset of Fig. 6c). This clearly signifies the induced FM order in CRO/STO film below 60 K. Above this temperature, the *ρ_xy_* is linear and resembles with that of CRO/LAO film. The value of R_0_ obtained for CRO/STO sample at 5 K is 4.76 × 10^−13^ Ω-cm/Oe or equivalent to 4.7653 × 10^−5^ cm^3^/C. Using this data, a simple one-band model gives a carrier concentration of ~1.31 × 10^23^ holes/cm^3^. While the carrier concentration derived for 200 K is ~0.27 × 10^23^ holes/cm^3^. In case of CRO/LAO, the carrier concentrations thus obtained is ~0.32 × 10^23^ holes/cm^3^ at 200 K and ~0.15 × 10^23^ electrons/cm^3^ at 5 K. These values confirm the sign reversal of the carrier polarity for CRO/LAO whereas no such sign reversal of charge carriers was observed in CRO/STO. Overall, these Hall data provide an unambiguous proof to magnetization data depicting intrinsic ferromagnetic order in tensile strained CRO/STO films and absence of the same in compressive strained CRO/LAO film.

**Figure 7:**
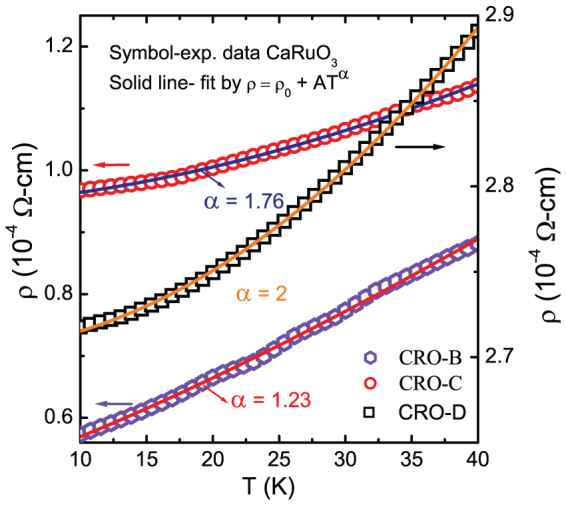
Fitting of the resistivity data of CRO-B, CRO-C and CRO-D films to the empirical relation *ρ* = *ρ*_0_ + *AT^α^*.

Non-magnetic CaRuO_3_ is a non-Fermi-liquid metal whereas its FM counterpart SrRuO_3_ exhibits a Fermi-liquid behavior. The magnetic ground state of CaRuO_3_ has been enigmatic for about past four decades. However, the present studies show that a FM order in this compound can be induced by manipulation of the structure. Now, to explore the consequent modifications in electronic properties of thus obtained FM CaRuO_3_ films, the temperature (T) dependence of resistivity (ρ) data were fitted to the empirical relation *ρ* = *ρ*_0_ + *AT^α^* where exponent α ~ 1.5 corresponds to non-Fermi liquid behavior and α ~ 2 corresponds to the Fermi-liquid behavior [Figure 7]. We found a clear relation between FM order and Fermi-liquid behavior in CaRuO_3_ films; the resistivity of non-magnetic CRO-B films with α ~ 1.23 is suggestive of near non-Fermi liquid behavior, whereas that of the FM CRO-D films shows α ~ 2 corresponding to the Fermi-liquid behavior. The resistivity of less ferromagnetic CRO-C films exhibits α ~ 1.76. These analyses clearly show that there is a gradual transition from a non-Fermi liquid to a Fermi-liquid behavior as we traverse from a non-magnetic to a ferromagnetic CaRuO_3_. In addition, there appears a clear similarity of the FM and Fermi-liquid behavior in CaRuO_3_ (CRO-D film) with the corresponding properties of well known SrRuO_3_.

## Discussion

There are several studies, mostly theoretical, on understanding the magnetic ground state of CaRuO_3_^10^,^11^,^20^,^26^,^27^,^28^. This is mainly because in bulk form it lacks any magnetic order whereas its magnetic analog SrRuO_3_ exhibits long range FM order. It was predicted that a FM order might set in CaRuO_3_ if by some appropriate means, chemical or physical, the tilt and rotation of RuO_6_ octahedra is reduced and the Ru-O-Ru bond distances and angles increased to match with those of the SrRuO_3_^11^,^27^. A manifestation of the same was unambiguously realized by chemical means, *i.e.*, via partial substitution of Ru both by magnetic ions^16^ as Cr or Fe and by non-magnetic ion^6^ as Ti. However, this technique has the drawback of transforming its metallic state to the semiconducting/insulating state. Zayak *et al* carried out detailed calculations of the correlations of epitaxial strain and magnetic moment in CaRuO_3_^20^ and suggested that a FM CaRuO_3_ may be formed by the means of inducing the tensile strain and in which the FM magnetic moment increases with the increasing tensile strain. Compressive strain, whereas, does not induce the same effect. An increase in in-plane Ru-O-Ru bond distances and a decrease in covalent character are two essential factors to induce magnetic order in CaRuO_3_, which can be achieved by tensile strain. A tensile strain of 2% can induce a saturation magnetic moment of ~0.5 μ_B_/f.u. In the present study, we find the experimental evidence to these theoretical predictions. It is clearly seen that a tensile strain of about 1% in CRO-C film induces a magnetic moment of ~0.06 μ_B_/f.u. and an enhanced strain of about 1.5% in CRO-D films results in a magnetic moment ~0.26 μ_B_/f.u. We could not form CaRuO_3_ films with higher tensile strain on substrate such as BaTiO_3_ (mismatch ~4%) and MgAl_2_O_4_ (mismatch ~6%). Indications of FM moment were found in pseudo-heterostructures of CaRuO_3_^29^. However, in such cases it is difficult to assign the occurrence of magnetic moment to a particular phase. A small magnetic moment in pseudo-heterostructures was attributed to the cubic phase which coexisted with relaxed and coherent orthorhombic phases. In comparison to this, the present studies show manifestation of an FM order with a clear magnetic transition in tensile strained orthorhombic CaRuO_3_ single-phase films. On comparing the FM properties of CaRuO_3_ films with those of the SrRuO_3_, we noted that the magnetic moment of the former is lower than that of the latter. However, the most noteworthy feature is a magnetic coercivity of ~200–300 Oe of CaRuO_3_ of CaRuO_3_ films is considerably lower than coercivity in the vicinity of 10 kOe for SrRuO_3_.

In summary, we have fabricated magnetic CaRuO_3_ films with single structural form and with varying tensile strain. We explicitly showed that FM moment in CaRuO_3_ can be induced by the means of tensile strain and that the magnitude of magnetic moment increases with the tensile strain. These observations are consistent with the theoretical predictions. We further show that tensile strain is more efficient that chemical route to induce magnetic order in CaRuO_3_. These leaves an intriguing aspect open: is it possible to induce larger magnetic moment of up to 1 μ_B_/f.u in CaRuO_3_ by adopting a combined approach of tensile epitaxial strain and optimal chemical substitutions? These studies also open up avenues to explore the utilization of tensile strained FM CaRuO_3_ vis-à-vis the FM SrRuO_3_ in spintronic and magnetic memory devices.

## Methods

### Sample preparation

Polycrystalline samples of CaRuO_3_ and CaRu_0.9_Cr_0.1_O_3_ were prepared by standard solid-state reaction route. The x-ray diffraction data confirmed the phase purity of both the samples. These samples were used for synthesis of thin films using pulsed laser deposition technique.

### Sample characterization

The θ-2θ diffraction patterns were collected by using PanAnalytical X-ray diffractrometer. The ω-2θ reciprocal space maps (RSM) using a four axis cradle mounted on same diffractrometer were also obtained for detailed structural and strain analyses of the films. The thickness of the samples was deduced from the reflectivity measurements. The magnetization measurements were performed using superconducting quantum interference device magnetometer. The resistivity and Hall measurements were performed using the four-probe and Van der Pauw geometry in Quantum Design PPMS system. The Hall resistivity ρ_xy_ was measured as a function of the applied magnetic field (H = ±9 T) at a constant temperature (5 K to 300 K).

## Author Contributions

D.S.R. conceived the project and designed the experiments in discussions with R.S.S., S.T. and R.R. S.T. and R.R. performed the syntheses and magnetization measurements. S.T., R.R., P.P. and S.K. analyzed the data. S.T. and S.K. performed the hall measurements. D.S.R., S.T., R.S.S. and S.K. wrote the paper. All authors contributed through scientific discussions.
